# Di-μ-aqua-bis­[aqua­(5-carboxyl­ato-1*H*-1,2,3-triazole-4-carb­oxy­lic acid-κ^2^ *N* ^3^,*O* ^4^)lithium]

**DOI:** 10.1107/S1600536813023167

**Published:** 2013-08-23

**Authors:** Wojciech Starosta, Janusz Leciejewicz

**Affiliations:** a Institute of Nuclear Chemistry and Technology, ul.Dorodna 16, 03-195 Warszawa, Poland

## Abstract

The crystal structure of the title compound, [Li_2_(C_4_H_2_N_3_O_4_)_2_(H_2_O)_4_], contains centrosymmetric dinuclear mol­ecules in which two Li^I^ ions are bridged by two water O atoms. The metal ion is coordinated by one *N*,*O*-bidentate ligand and three water O atoms (one of them is symmetry generated), with one of the bridging water O atoms in the apical position of a distorted square pyramid. The carboxyl­ate group that participates in coordination to the metal ion remains protonated; the other is deprotonated and coordination inactive. An intra­molecular O—H⋯O hydrogen bond between carboxyl­ate groups is observed. In the crystal, dimers are linked by O—H⋯O, O—H⋯N and N—H⋯O hydrogen bonds, generating a three-dimensional network.

## Related literature

For the structures of Co and Ni complexes with the 1,2,3-trizole-4,5-di­carboxyl­ate ligand, see: Tong *et al.* (2011 ▶).
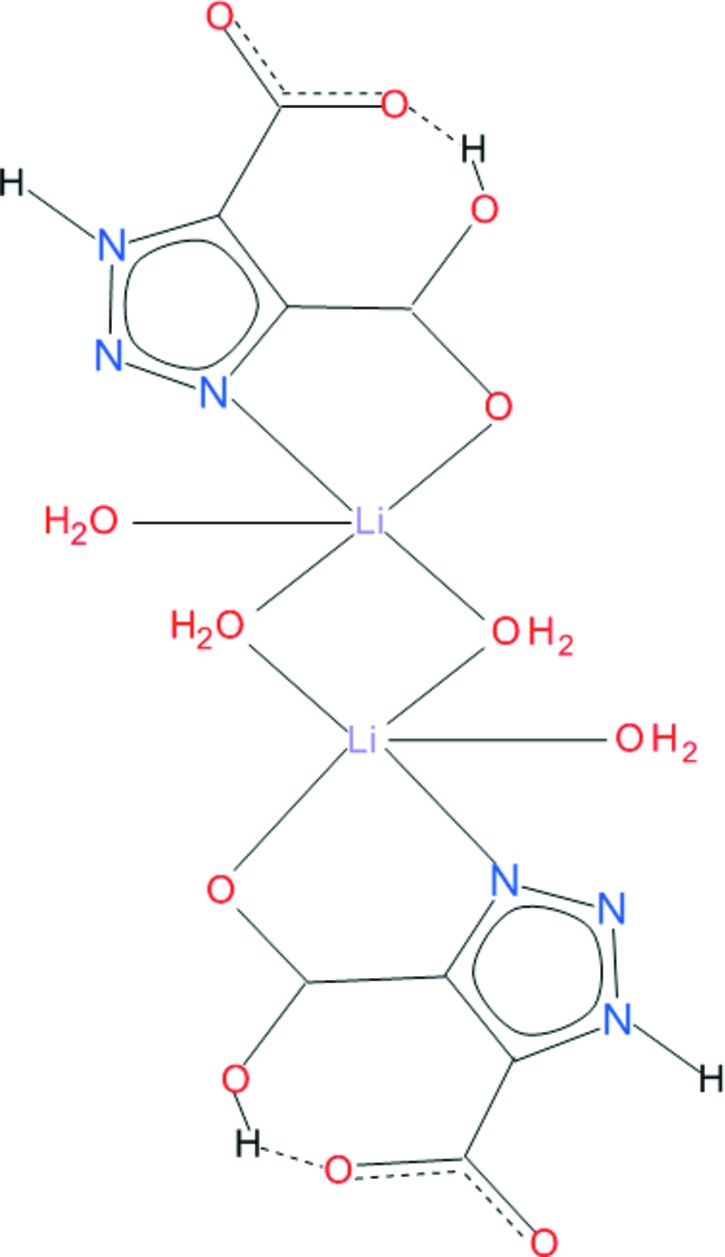


## Experimental

### 

#### Crystal data

[Li_2_(C_4_H_2_N_3_O_4_)_2_(H_2_O)_4_]
*M* *_r_* = 398.12
Triclinic, 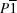

*a* = 5.1264 (10) Å
*b* = 8.0350 (16) Å
*c* = 10.040 (2) Å
α = 68.60 (3)°
β = 77.64 (3)°
γ = 85.16 (3)°
*V* = 376.12 (13) Å^3^
*Z* = 1
Mo *K*α radiation
μ = 0.16 mm^−1^
*T* = 293 K
0.45 × 0.26 × 0.22 mm

#### Data collection

Kuma KM-4 four-circle diffractometer
Absorption correction: analytical (*CrysAlis RED*; Oxford Diffraction, 2008 ▶) *T*_min_ = 0.951, *T*_max_ = 0.966
2414 measured reflections
2185 independent reflections
1695 reflections with *I* > 2σ(*I*)
*R*_int_ = 0.110
3 standard reflections every 200 reflections intensity decay: 3.7%

#### Refinement

*R*[*F* ^2^ > 2σ(*F* ^2^)] = 0.057
*wR*(*F* ^2^) = 0.151
*S* = 1.04
2185 reflections
151 parameters
All H-atom parameters refined
Δρ_max_ = 0.62 e Å^−3^
Δρ_min_ = −0.43 e Å^−3^

### 

Data collection: *KM-4 Software* (Kuma, 1996 ▶); cell refinement: *KM-4 Software*; data reduction: *DATAPROC* (Kuma, 2001 ▶); program(s) used to solve structure: *SHELXS97* (Sheldrick, 2008 ▶); program(s) used to refine structure: *SHELXL97* (Sheldrick, 2008 ▶); molecular graphics: *SHELXTL* (Sheldrick, 2008 ▶); software used to prepare material for publication: *SHELXTL*.

## Supplementary Material

Crystal structure: contains datablock(s) I, New_Global_Publ_Block. DOI: 10.1107/S1600536813023167/hb7130sup1.cif

Structure factors: contains datablock(s) I. DOI: 10.1107/S1600536813023167/hb7130Isup2.hkl

Additional supplementary materials:  crystallographic information; 3D view; checkCIF report

## Figures and Tables

**Table 1:** Selected bond lengths (Å)

Li1—O1	2.156 (3)
Li1—O5	2.089 (3)
Li1—O5^i^	2.029 (3)
Li1—O6	1.916 (3)
Li1—N3	2.234 (3)

Symmetry code: (i) 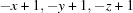.

**Table 2:** Hydrogen-bond geometry (Å, °)

*D*—H⋯*A*	*D*—H	H⋯*A*	*D*⋯*A*	*D*—H⋯*A*
O5—H52⋯O1^ii^	0.86 (2)	1.93 (2)	2.7873 (16)	172 (2)
O6—H61⋯N2^iii^	0.84 (4)	2.30 (4)	3.0545 (19)	150 (4)
O5—H51⋯O3^iv^	0.90 (2)	1.98 (3)	2.8834 (16)	174 (2)
O6—H62⋯O4^v^	0.91 (5)	1.83 (5)	2.7343 (16)	176 (4)
N1—H1⋯O4^vi^	0.86 (2)	1.92 (2)	2.7522 (17)	162 (2)
O2—H2⋯O3	0.93 (5)	1.63 (5)	2.5380 (16)	167 (4)

Symmetry codes: (ii) 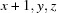; (iii) 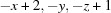; (iv) 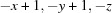; (v) 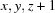; (vi) 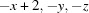.

**Fig. 1.**
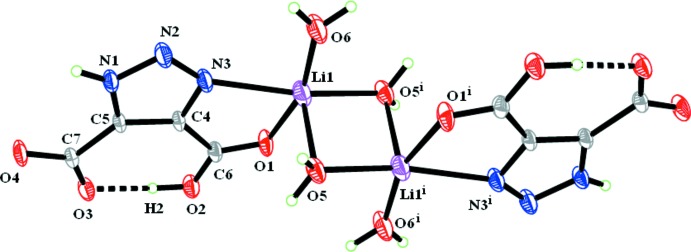
A dimeric molecule of the title complex with 50% probability displacement ellipsoids. Symmetry code: (i): -x + 1, -y + 1, -z + 1.

**Fig. 2.**
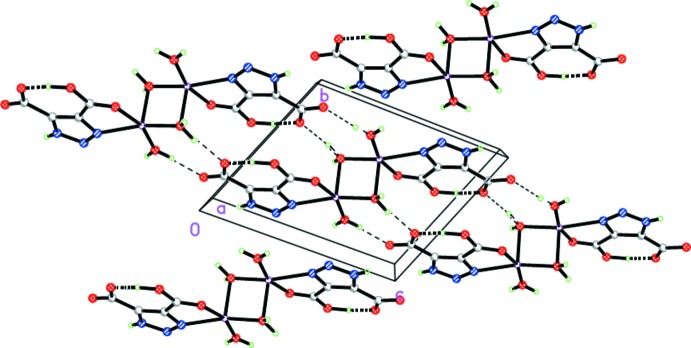
The packing of the dimers with hydrogen bonds shown as dashed lines.

## supplementary crystallographic information

### 1. Comment

The triclinic unit cell of the title compound comprises two Li(C_4_H_2_N_3_O_4_)(H_2_O)_2_ molecules related by an inversion centre to form a dimeric moiety in which two Li^I^ ions are bridged by an aqua O atom donated by each molecule {Fig.1). The coordination of the Li^I^ ion is distorted square-pyramidal: carboxylate O1, hetero-ring N1, aquua O6 and O5^(i)^ atoms constitute its base [r.m.s. 0.0798 (2) Å], the Li1 ion is 0.1995 (2) Å out of it, the aqua O5^(i)^ is at the apex. Li—O and Li—N bond distances are usual (Table 2). The ligand triazole ring is almost planar [r.m.s. 0.0006 (1) Å]. The carboxylate C6/O1/O2 and C7/O3/O4 groups make with it dihedral angles of 2.0 (1)° and 5.5 (1)°, respectively. The Fourier map indicates clearly that the O2 atom is protonated and acts as a donor in a fairly short intra-molecular hydrogen bond of 2.538 (2) Å to the O3 atom as an acceptor. The C7/O3/O4 carboxylic group remains deprotonated and coordination inactive. The bond distances and bond angles within the triazole ring do not differ from those reported in the structures of other complexes, for example, with Co and Ni (Tong *et al.*, 2011). The dimers form molecular sheets (Fig. 2) in which they interact *via* an extensive hydrogen bond network; coordinated water molecules are as donors a hetero-ring N atom and carboxylate O atoms as acceptors (Table 3).

### 2. Experimental

1 mmol of 1,2,3-triazole-4,5-dicarboxylic acid and *ca*2 mmol s of lithium hydroxide dissolved in 50 ml of hot, doubly distilled water were boiled under reflux with stirring for ten hours and then left to crystallize at room temperature. Colourless blocks deposited after a week among polycrystalline material. After extraction, the crystals were washed with cold ethanol and dried in the air.

### 3. Refinement

H atoms belonging to water molecules, the carboxylate group and hetero-ring N atom were located in a difference map and refined isotropically.

### Crystal data

**Table:**

[Li_2_(C_4_H_2_N_3_O_4_)_2_(H_2_O)_4_]	*Z* = 1
*M**_r_* = 398.12	*F*(000) = 204
Triclinic, *P*1	*D*_x_ = 1.758 Mg m^−^^3^
Hall symbol: -P 1	Mo *K*α radiation, λ = 0.71073 Å
*a* = 5.1264 (10) Å	Cell parameters from 25 reflections
*b* = 8.0350 (16) Å	θ = 6–15°
*c* = 10.040 (2) Å	µ = 0.16 mm^−^^1^
α = 68.60 (3)°	*T* = 293 K
β = 77.64 (3)°	Blocks, colourless
γ = 85.16 (3)°	0.45 × 0.26 × 0.22 mm
*V* = 376.12 (13) Å^3^	

### Data collection

**Table:**

Kuma KM-4 four-circle diffractometer	1695 reflections with *I* > 2σ(*I*)
Radiation source: fine-focus sealed tube	*R*_int_ = 0.110
Graphite monochromator	θ_max_ = 30.1°, θ_min_ = 2.2°
profile data from ω/2θ scan	*h* = 0→7
Absorption correction: analytical (*CrysAlis RED*; Oxford Diffraction, 2008)	*k* = −11→11
*T*_min_ = 0.951, *T*_max_ = 0.966	*l* = −13→13
2414 measured reflections	3 standard reflections every 200 reflections
2185 independent reflections	intensity decay: 3.7%

### Refinement

**Table:**

Refinement on *F*^2^	Primary atom site location: structure-invariant direct methods
Least-squares matrix: full	Secondary atom site location: difference Fourier map
*R*[*F*^2^ > 2σ(*F*^2^)] = 0.057	Hydrogen site location: inferred from neighbouring sites
*wR*(*F*^2^) = 0.151	All H-atom parameters refined
*S* = 1.04	*w* = 1/[σ^2^(*F*_o_^2^) + (0.121*P*)^2^] where *P* = (*F*_o_^2^ + 2*F*_c_^2^)/3
2185 reflections	(Δ/σ)_max_ < 0.001
151 parameters	Δρ_max_ = 0.62 e Å^−^^3^
0 restraints	Δρ_min_ = −0.43 e Å^−^^3^

### Special details

**Table:**

Geometry. All e.s.d.'s (except the e.s.d. in the dihedral angle between two l.s. planes) are estimated using the full covariance matrix. The cell e.s.d.'s are taken into account individually in the estimation of e.s.d.'s in distances, angles and torsion angles; correlations between e.s.d.'s in cell parameters are only used when they are defined by crystal symmetry. An approximate (isotropic) treatment of cell e.s.d.'s is used for estimating e.s.d.'s involving l.s. planes.
Refinement. Refinement of *F*^2^ against ALL reflections. The weighted *R*-factor *wR* and goodness of fit *S* are based on *F*^2^, conventional *R*-factors *R* are based on *F*, with *F* set to zero for negative *F*^2^. The threshold expression of *F*^2^ > σ(*F*^2^) is used only for calculating *R*-factors(gt) *etc*. and is not relevant to the choice of reflections for refinement. *R*-factors based on *F*^2^ are statistically about twice as large as those based on *F*, and *R*- factors based on ALL data will be even larger.

### Fractional atomic coordinates and isotropic or equivalent isotropic displacement parameters (Å^2^)

**Table:**

	*x*	*y*	*z*	*U*_iso_*/*U*_eq_	
O1	0.21219 (19)	0.40701 (15)	0.33815 (11)	0.0281 (2)	
N3	0.6270 (2)	0.17626 (16)	0.33674 (12)	0.0254 (3)	
C5	0.6283 (2)	0.17546 (15)	0.11571 (12)	0.0194 (2)	
C4	0.5041 (2)	0.24296 (16)	0.22126 (12)	0.0195 (3)	
N1	0.8213 (2)	0.07012 (16)	0.17492 (12)	0.0245 (3)	
C6	0.2768 (2)	0.36533 (16)	0.22992 (13)	0.0205 (3)	
N2	0.8203 (2)	0.07056 (17)	0.30842 (13)	0.0295 (3)	
O3	0.38723 (19)	0.29832 (14)	−0.07107 (10)	0.0269 (2)	
O4	0.7499 (2)	0.13549 (15)	−0.10818 (11)	0.0301 (3)	
O5	0.71001 (19)	0.56809 (15)	0.36456 (11)	0.0280 (2)	
O6	0.7261 (3)	0.19431 (19)	0.60872 (13)	0.0419 (3)	
Li1	0.4924 (5)	0.3334 (4)	0.4828 (3)	0.0317 (5)	
H52	0.871 (4)	0.527 (3)	0.358 (2)	0.032 (5)*	
H51	0.682 (4)	0.618 (3)	0.272 (3)	0.042 (6)*	
O2	0.15267 (19)	0.42563 (14)	0.12078 (11)	0.0266 (2)	
C7	0.5863 (2)	0.20502 (16)	−0.03313 (12)	0.0207 (3)	
H61	0.848 (8)	0.120 (5)	0.600 (4)	0.094 (12)*	
H62	0.735 (8)	0.181 (6)	0.701 (5)	0.117 (14)*	
H2	0.214 (8)	0.380 (6)	0.047 (5)	0.099 (12)*	
H1	0.944 (4)	0.012 (3)	0.136 (2)	0.036 (5)*	

### Atomic displacement parameters (Å^2^)

**Table:**

	*U*^11^	*U*^22^	*U*^33^	*U*^12^	*U*^13^	*U*^23^
O1	0.0267 (4)	0.0409 (6)	0.0228 (5)	0.0088 (4)	−0.0017 (3)	−0.0222 (4)
N3	0.0307 (5)	0.0320 (6)	0.0160 (5)	0.0076 (4)	−0.0041 (4)	−0.0134 (4)
C5	0.0236 (5)	0.0229 (5)	0.0130 (5)	0.0028 (4)	−0.0005 (4)	−0.0104 (4)
C4	0.0236 (5)	0.0232 (5)	0.0131 (5)	0.0020 (4)	0.0000 (4)	−0.0106 (4)
N1	0.0300 (5)	0.0298 (5)	0.0163 (5)	0.0098 (4)	−0.0044 (4)	−0.0135 (4)
C6	0.0205 (5)	0.0248 (6)	0.0170 (5)	0.0014 (4)	0.0014 (4)	−0.0117 (4)
N2	0.0367 (6)	0.0372 (6)	0.0188 (5)	0.0135 (5)	−0.0091 (4)	−0.0159 (5)
O3	0.0302 (5)	0.0359 (5)	0.0171 (4)	0.0086 (4)	−0.0051 (3)	−0.0138 (4)
O4	0.0360 (5)	0.0401 (6)	0.0175 (4)	0.0134 (4)	−0.0031 (4)	−0.0184 (4)
O5	0.0258 (5)	0.0406 (6)	0.0181 (4)	0.0071 (4)	−0.0011 (3)	−0.0144 (4)
O6	0.0534 (7)	0.0546 (7)	0.0232 (5)	0.0265 (5)	−0.0144 (5)	−0.0224 (5)
Li1	0.0375 (12)	0.0382 (13)	0.0210 (11)	0.0030 (10)	−0.0014 (9)	−0.0158 (10)
O2	0.0275 (4)	0.0341 (5)	0.0215 (5)	0.0086 (3)	−0.0050 (3)	−0.0155 (4)
C7	0.0259 (5)	0.0241 (5)	0.0132 (5)	0.0016 (4)	−0.0006 (4)	−0.0102 (4)

### Geometric parameters (Å, º)

**Table:**

Li1—O1	2.156 (3)	N1—N2	1.3405 (15)
Li1—O5	2.089 (3)	N1—H1	0.86 (2)
Li1—O5^i^	2.029 (3)	C6—O2	1.3035 (16)
Li1—O6	1.916 (3)	O3—C7	1.2559 (16)
Li1—N3	2.234 (3)	O4—C7	1.2415 (15)
O1—C6	1.2229 (15)	O5—Li1^i^	2.029 (3)
N3—N2	1.3029 (16)	O5—H52	0.86 (2)
N3—C4	1.3529 (16)	O5—H51	0.90 (2)
C5—N1	1.3386 (17)	O6—H61	0.84 (4)
C5—C4	1.3765 (16)	O6—H62	0.91 (5)
C5—C7	1.4852 (16)	Li1—Li1^i^	2.831 (5)
C4—C6	1.4692 (17)	O2—H2	0.93 (5)
			
C6—O1—Li1	115.52 (11)	Li1—O6—H61	133 (3)
N2—N3—C4	108.64 (11)	Li1—O6—H62	132 (3)
N2—N3—Li1	140.62 (11)	H61—O6—H62	95 (4)
C4—N3—Li1	108.55 (11)	O6—Li1—O5^i^	92.37 (11)
N1—C5—C4	103.63 (11)	O6—Li1—O5	102.05 (13)
N1—C5—C7	123.52 (11)	O5^i^—Li1—O5	93.17 (11)
C4—C5—C7	132.82 (11)	O6—Li1—O1	161.54 (16)
N3—C4—C5	108.86 (11)	O5^i^—Li1—O1	98.57 (11)
N3—C4—C6	118.02 (11)	O5—Li1—O1	92.20 (12)
C5—C4—C6	133.12 (12)	O6—Li1—N3	89.77 (11)
C5—N1—N2	111.56 (11)	O5^i^—Li1—N3	165.21 (15)
C5—N1—H1	128.6 (14)	O5—Li1—N3	100.70 (11)
N2—N1—H1	119.7 (14)	O1—Li1—N3	75.95 (9)
O1—C6—O2	122.70 (12)	O6—Li1—Li1^i^	100.59 (15)
O1—C6—C4	119.06 (12)	O5^i^—Li1—Li1^i^	47.47 (8)
O2—C6—C4	118.23 (11)	O5—Li1—Li1^i^	45.70 (8)
N3—N2—N1	107.31 (11)	O1—Li1—Li1^i^	97.76 (14)
Li1^i^—O5—Li1	86.83 (11)	N3—Li1—Li1^i^	146.10 (17)
Li1^i^—O5—H52	126.9 (15)	C6—O2—H2	115 (3)
Li1—O5—H52	101.1 (13)	O4—C7—O3	126.12 (12)
Li1^i^—O5—H51	118.6 (15)	O4—C7—C5	117.29 (12)
Li1—O5—H51	112.5 (13)	O3—C7—C5	116.59 (11)
H52—O5—H51	107 (2)		

Symmetry code: (i) −*x*+1, −*y*+1, −*z*+1.

### Hydrogen-bond geometry (Å, º)

**Table:**

*D*—H···*A*	*D*—H	H···*A*	*D*···*A*	*D*—H···*A*
O5—H52···O1^ii^	0.86 (2)	1.93 (2)	2.7873 (16)	172 (2)
O6—H61···N2^iii^	0.84 (4)	2.30 (4)	3.0545 (19)	150 (4)
O5—H51···O3^iv^	0.90 (2)	1.98 (3)	2.8834 (16)	174 (2)
O6—H62···O4^v^	0.91 (5)	1.83 (5)	2.7343 (16)	176 (4)
N1—H1···O4^vi^	0.86 (2)	1.92 (2)	2.7522 (17)	162 (2)
O2—H2···O3	0.93 (5)	1.63 (5)	2.5380 (16)	167 (4)

Symmetry codes: (ii) *x*+1, *y*, *z*; (iii) −*x*+2, −*y*, −*z*+1; (iv) −*x*+1, −*y*+1, −*z*; (v) *x*, *y*, *z*+1; (vi) −*x*+2, −*y*, −*z*.
